# The anticancer potential of tetrahydrocurcumin-phytosomes against oral carcinoma progression

**DOI:** 10.1186/s12903-024-04856-9

**Published:** 2024-09-26

**Authors:** Nehal Raouf, Zeinab Elsayed Darwish, Omneya Ramadan, Hebatallah S. Barakat, Shimaa A. Elbanna, Marwa M. Essawy

**Affiliations:** 1 Department of Oral Pathology, Faculty of Dentistry, Alexandria University, Champollion Street, Elazarita, Alexandria, 21563 Egypt; 2 Department of Pharmaceutics, Faculty of Pharmacy, Alexandria University, Alexandria, Egypt; 3 Center of Excellence for Research in Regenerative Medicine and its Applications (CERRMA), Faculty of Medicine, Alexandria University, Alexandria, Egypt

**Keywords:** Bax, Caspase, Mitotracker Red, Squamous cell carcinoma, S-phase

## Abstract

**Background:**

Herbal medicine combined with nanotechnology offers an alternative to the increasing burden of surgery and/or chemotherapy, the main therapeutics of oral carcinoma. Phytosomes are nano-vesicular systems formed by the interaction between phospholipids and phyto-active components via hydrogen bonding, exhibiting superior efficacy over pure phytocomponents in drug delivery.

**Methods:**

Tetrahydrocurcumin (THC)-phytosomes were prepared by thin film hydration method. After characterization, in vitro cytotoxicity, antiproliferative capacity, antioxidant potential and full apoptotic workup were paneled on oral squamous cell carcinoma (SCC4) in comparison with native THC-solution and cisplatin (3.58 µg/mL intravenous injection), as positive controls. In addition, we tested the three medications on normal oral keratinocytes and gingival fibroblasts to attest to their tissue-selectivity.

**Results:**

Successful preparation of THC-phytosomes using 1:1 molar ratio of THC to phospholipid exhibited significantly increased aqueous solubility, good colloidal properties, and complete drug release after one hour. On SCC4 cells, THC-phytosomes, at their dose-/time-dependency at ~ 60.06 µg/mL escalated cell percentages in the S-phase with 32.5 ± 6.22% increase, as well as a startling 29.69 ± 2.3% increase in apoptotic population. Depletion of the cell colonies survival to 0.29 ± 0.1% together with restraining the migratory rate by -6.4 ± 6.8% validated THC-phytosomes’ antiproliferative capacity. Comparatively, the corresponding results of THC-solution and cisplatin revealed 12.9 ± 0.9% and 25.8 ± 1.1% for apoptosis and 0.9 ± 0.1% and 0.7 ± 0.08% for colony survival fraction, respectively. Furthermore, the nanoformulation exhibited the strongest immuno-positivity to caspase-3, which positively correlated with intense mitochondrial fluorescence by Mitotracker Red, suggesting its implication in the mitochondrial pathway of apoptosis, a finding further explained by the enormously high *Bax* and *caspase-8* expression by RT-qPCR. Finally, the THC groups showed the lowest oxidative stress index, marking their highest free radical-scavenging potential among the test groups.

**Conclusions:**

THC-phytosomes are depicted to be an efficient nanoformulation that enhanced the anticancer efficacy over the free drug counterpart and the conventional chemotherapeutic. Additionally, being selective to cancer cells and less cytotoxic to normal cells makes THC-phytosomes a potential candidate for tissue-targeted therapy.

**Supplementary Information:**

The online version contains supplementary material available at 10.1186/s12903-024-04856-9.

## Background

Nanotechnology is among the approaches tackled nowadays to overcome the obstacles faced in oral cancers management [1], the 6th most common cancer globally when combined with oropharyngeal cancer [2]. Accounting for more than 90% of those cancers is oral squamous cell carcinoma (OSCC) [3]. OSCC is a malignancy of epithelial origin with a complex multifactorial etiology. Habitual factors such as smoking (tobacco, cigarettes or areca nut) and alcohol consumption play an important role in disease progression. Genetics and microbial factors, which often vary geographically and ethnically, are among other factors [4]. A majority of OSCC cases also arise from oral precancerous lesions, which are altered tissues morphologically and histologically, that often present as white patches (leukoplakia) or red patches (erythroplakia) [5]. OSCC is currently managed by surgery, radiotherapy and/or chemotherapy [6]. However, drug resistance, systemic toxicity, and limited therapeutic effect are major limitations that face clinicians [7]. In addition, oral complications of patients undergoing chemo/radiotherapies are frequent, distressing, and largely affect the quality of life [8]. Herbal medicine, combined with nanotechnology, is currently one of the most interesting topics of research for alternatives or adjuvants to conventional chemotherapeutics in the treatment of OSCC [9]. Curcumin, derived from curcuma longa, is one of the most famous herbal extracts known for a wide range of anticancer attributes, however, it is notorious for low bioactivity, solubility, and chemical stability, which cause low uptake into cancer cells [10]. Tetrahydrocurcumin (THC), an isolated colorless major metabolite of curcumin, is a polyphenolic compound that holds a special interest since it shows better chemical stability and bioavailability than its parent compound [11]. THC exhibits curcumin-like pharmacokinetic properties, with exceptional antioxidant potential [12]. Some studies even assert that THC, among other isolated metabolites of curcumin, are the ones responsible for the health benefits provided by the herb itself [13]. It possesses exemplary chemo-preventive and therapeutic potential against a wide range of malignancies [14–16]. THC shows an array of molecular targets such as an antiproliferative activity through decreasing p21 protein [17], having an antioxidative potential [18], inducing apoptosis and promoting natural killer cell activity and phagocytosis [14, 19].

Despite its upper hand in chemical stability over curcumin [20], THC still shows low aqueous solubility that limits its bio-accessibility [21]. Therefore, several drug delivery systems have been developed till date to transport THC to enhance its efficiency, improve solubility, and reach improved drug uptake. Among those are optimized self-nanoemulsifying drug delivery systems [22], 3D-printed mucoadhesive collagen scaffolds [23], nanoemulsions [24], chitosan composites [25], lipid nanocarriers [26], gold-alloy nanocomposites [27], and nanocrystals [28].

Phytosomes (phyto-phospholipid complexes), liposomes and transferosomes are examples of lipid-based organic delivery systems, which have the potential to increase the concentration of poorly soluble drugs, their absorption, and stability. Structurally, the phytochemical (drug) is mixed with the phospholipid to form an integrated complex known as the phytosome. In case of liposomes or transferosomes, the phytochemical is engulfed within the core [29]. This structural difference in the phytosome is due to the formation of hydrogen bonds between the polar head of the phospholipid and the polar group of the drug [30]. These bonds enhance the efficiency of drug entrapment, water solubility, and minimizes drug leakage. This, in turn, improves drug stability, increases membrane absorption, conclusively maximizing bioavailability [31].

Our previous in vivo hamster oral carcinogenic model demonstrated the effectiveness of a locally administered nanocomposite sponge loaded with THC in managing oral precancerous leukoplakic lesions [28]. In the current work, we aim to develop THC loaded phytosomes as a unique nanoformulation with enhanced anticancer effect against oral cancer. Characterization of THC-phytosomes and determination of their cytotoxic potential against human oral squamous cell carcinoma cell line (SCC4) as well as their selectivity towards normal cell lines were pursued. Additionally, several molecular targets and signaling pathways of THC-phytosomes were investigated and compared with free THC-solution and cisplatin as a positive control. The combined findings of this study provide affirmation of the potency of THC-phytosomes as an anti-apoptotic, antioxidant, and antiproliferative nanoformulation over its free native counterpart and the chemotherapeutic agent.

## Methods

### Drugs, reagents, and cell culture

The used drugs and reagents are detailed in the supplementary material. The human oral cancer cell line SCC4 was obtained from the American Type Culture Collection (ATCC, Virginia, US). Human gingival fibroblasts and oral epithelial cells were isolated and donated by the Center of Excellence for Research in Regenerative Medicine and Application (CERRMA).

### Preparation of THC-phytosomes

THC-phytosomes were prepared by the thin-film hydration method [31], using three molar ratios of THC to the phospholipid, soy phosphatidylcholine (SPC) (Table 1). THC and SPC (Lipoid^®^ S100) were dissolved in absolute ethanol which was then evaporated under vacuum at 45 °C to produce THC-phytosomes waxy compound. THC-phytosomes aqueous dispersion was obtained by hydrating the resulting waxy compound using distilled water at room temperature. THC-phytosomes were subsequently characterized. Some of the characterization tests were performed on the THC-phytosomes waxy compound to assess the impact of SPC complexation on THC physicochemical properties. Other tests were performed on the THC-phytosomes aqueous dispersion to investigate the colloidal properties, in vitro drug release profile, and morphology.

**Table 1:** Composition and characterization parameters of different THC-phytosomes formulations compared to pure THC

Formulation code	THC: SPC Molar ratio	Particle size (nm)	Polydispersity index	Zeta potential (mV)	Solubility in water (mg %)	Partition coefficient (*P*_o/w_)
Pure THC	**-**	**-**	**-**	**-**	3 ± 0.01	11 ± 0.02
P1	1:1	317.35 ± 5.48	0.285 ± 0.022	-29.88 ± 0.351	25.7 ± 0.01	7 ± 0.01
P2	1:2	419.08 ± 20.68	0.637 ± 0.190	-30.46 ± 0.412	13 ± 0.01	9.5 ± 0.2
P3	1:3	442.11 ± 16.31	0.641 ± 0.036	-32.21 ± 0.366	13 ± 0.02	10 ± 0.1

### Characterization of THC-phytosomes

Characterization was performed to select the optimum THC-phytosomes formulation for subsequent studies.

#### Solubility study

It was conducted to assess the impact of SPC complexation on THC’s water solubility. The apparent solubility of the three THC-phytosomes waxy compounds (THC: SPC; 1:1, 1:2, and 1:3) was determined and compared to that of pure THC using the shake-flask method [32]. Briefly, excess THC or THC-phytosomes waxy compounds was added to 2 ml of water in sealed glass vials, shaken at 25 °C, 100 rpm for 24 h. and then centrifuged at 4000 rpm for 15 min to remove excess insoluble drug. The withdrawn supernatant was filtered through a 0.45 μm filter and diluted with methanol for THC quantification at 281 nm by a UV-spectrophotometer (UV-160; Shimadzu).

The withdrawn samples were diluted with methanol for THC quantification at 281 nm by a UV-spectrophotometer (UV-160; Shimadzu).

#### Partition coefficient (Po/w)

Characteristics of THC and different THC-phytosomes waxy compounds were obtained employing the shake-flask method [33, 34]. 1 mL n-octanol (pre-saturated by water) was added to 1 mL of saturated water solution of THC and THC-phytosomes into 5 mL test tubes and then shaken at 25 °C, 100 rpm for 24 h. Afterwards, the sample was centrifuged at 4000 rpm for 15 min at 25 °C. THC concentration in both layers was determined using UV spectrometer at λ max 281 nm. Consequently, P _o/w_ was calculated from Eq. 1:

1 $$\:P(o/w)=\frac{Cn}{Cw},$$

where Cn and Cw were the THC concentrations in n-octanol and water, respectively.

#### Colloidal properties assessment

Particle size, polydispersity index, and zeta potential were assessed for the freshly prepared THC-phytosomes aqueous dispersions (30-fold diluted) using Malvern Zetasizer (Malvern Instruments, UK) to select the optimum formulation.

#### In vitro drug release study

Dialysis method was used to evaluate THC release profiles from different THC-phytosomes aqueous dispersions compared to THC suspension in ethanol 50% v/v under sink condition at 37 ± 0.5 °C. Samples were withdrawn at (0, 1, 2 and 3 h). Samples were analyzed spectrophotometrically at 280 nm against release medium blank.

#### Transmission Electron Microscopy (TEM)

It was performed to visualize the morphology of the selected THC-phytosomes aqueous dispersion (P1) using (Jeol, JEM-100 CX electron microscope (Japan) [35].

#### Fourier Transform Infrared Spectroscopy (FTIR)

It was used to record the spectra for THC, SPC, and the selected P1 waxy compound using spectrometer (Cary 630, Agilent, USA). Samples were scanned between 700 and 4000 cm − 1. Spectra were recorded at a spectral resolution of 2 cm-1 with an average of 20 scans.

#### Differential Scanning Calorimetry (DSC)

It was used to evaluate the thermal behaviors of THC, SPC, and P1 using DSC-6 (Shimadzu, Tokyo, Japan). The sealed samples were heated from 25 to 200 °C at a constant rate of 10 °C/min under nitrogen atmosphere (60 mL/min).

#### Stability study

It was performed for the selected THC-phytosomes (P1) during storage at 4 °C for 4 months. Stability of its colloidal properties after hydration was assessed at one-month intervals for 4 months.

### Cytotoxicity and selectivity index

Briefly, cells (cancerous and normal) in 96-well plates were seeded at a density of 5000 cells/well in 100 µL DMEM. After 24 h-incubation, cells were treated with serial concentrations of THC-phytosomes (40–160 µg/mL) and THC-solution (50–350 µg/mL, positive control), taking cisplatin (0.5–22.5 µg/mL) as another positive control to be compared with untreated SCC4 cells (negative control). Then, MTT was added at the predetermined period (24 and 48 h), and its absorbance was detected using ELISA microplate reader (Infinite F15 TECAN, Switzerland) [36]. Viability % and selectivity index were then calculated (Suppl. Material).

### Clonogenic survival assay

Clonogenic survival assay was performed to evaluate the long-term survival and proliferation potential of the treated oral cancer cells. In 6-well plates, 1000 cells were seeded. Fourteen days later, oral cancer cells were incubated with the determined IC50 of the proposed treatments for 48 h. Then, the cells were fixed with 4% neutral buffered formalin and stained with crystal violet. Colonies with more than 50 cells were counted using ImageJ (1.54f, NIH, USA). We then calculated the plating efficiency (PE) and survival fraction [37]. (Suppl. Material)

### Wound healing assay

After obtaining a confluent monolayer of cells, a 200 µL sterile pipette tip was used to inflict a scratch line, then cells were PBS-rinsed. After different treatments, the open wound width was measured and photomicrographed using a phase contrast microscope (Olympus BX41) at 0, 24, 48 and 72 h time intervals. (Suppl. Material)

### Flow cytometric analysis of apoptosis and cell cycle arrest

After adding the proposed treatments, cells were obtained, washed with PBS, and stained with Annexin V-FITC followed by PI. FACScan flow cytometry (BD, Bioscience, US) was used to assess the cell cycle phases and fluorescence of stained cells [37].

### Cytological visualization

To assess the nuclear and cytoplasmic changes after drug administration, we took a smear from the treated SCC4 cells. After trypsinization, treated cells in a 6-well plate were fixed with formaldehyde and processed for H&E staining protocols. The examination was done at ×1000 through an Olympus DP20 digital camera attached to the Olympus BX41 microscope [38].

### Immunohistochemistry

Formaldehyde-fixed, paraffin-embedded SCC4 cells were processed with standard immunohistochemical (IHC) staining with (1:1000) caspase-3 antibody according to the manufacturer’s protocol.

### Mitochondrial assessment during apoptosis

After treating cancer cells -seeded (2 × 10^5^ cells/well) on cover slip in 6-well plates- with the proposed treatments for 48 h, 100 nM MitoTracker Red were added and incubated for 1 h in a CO2 incubator at 37 °C. Prior to fluorescence imaging, nuclear staining was done following the steps in the next section. Visualization was done using a confocal microscope Leica DMi8 (Leica, Wetzlar, Germany). The images were captured and analyzed using ImageJ (1.54f, NIH, USA). (Suppl. Material)

### Appraisement of apoptosis with Hoechst staining

To demonstrate the nuclear apoptotic features, SCC4 cells (5 × 10^5^ cells/well) were seeded into a 6-well plate with cover glass and incubated overnight in DMEM high glucose of 10% FBS and 1% P/S at 5% CO_2_ and 37 °C. Following treatment with the IC50s of the three medications, the cells were twice washed with PBS, fixed with paraformaldehyde (4%), permeabilized with Triton X-100 (0.1%), then stained with Hoechst 33,342 dye solution (1 µg/mL), and finally examined using confocal microscope Leica DMi8 (Leica, Wetzlar, Germany). The images were captured and analyzed using ImageJ (1.54f, NIH, USA) [39].

### Reverse transcription quantitative polymerase chain reaction (RT-qPCR)

Following the standard procedures of isolation and reverse transcription of RNA from different treated SCC4 plated on 6-well plates, real time PCR was performed using the relative quantification (ΔΔCt) method (Suppl. Material). Primer sequences were as follows:

#### Caspase-8

F5′-CCTGGGTGCGTCCACTTT-3′,

R 5ʹ- CAAGGTTCAAGTGACCAACTCAAG-3′.

#### Bax

F 5ʹ-TTCATCCAGGATCGAGCAG-3ʹ,

R 5ʹ-TGAGACACTCGCTCAGCTTC-3′.

#### HPRT

F 5′-TGACACTGGCAAAACAAT-3′,

R 5′-GGTCCTTTTCACCAGCAA-3′.

### Oxidative stress biomarkers assay

Oxidative stress was assessed in the culture media by measuring the ratio of the lipid peroxidation (LPO) product malondialdehyde (MDA) to total antioxidant capacity (TAC) after administering the IC50 of the three medications (Suppl. Material). The ratio of MDA/TAC was calculated by dividing the MDA (nmol/mL) value by the TAC (mM/L) [40].

### Statistical analysis

All the experiments were carried out in three independent experiments each of triplicate and the results were declared as the mean ± SD after analysis by GraphPad Prism (9.5.1 GraphPad Software, San Diego, California USA). A *p-*value < 0.05 was considered statistically significant. The characterization results were analyzed using Student’s t-test. The non-linear regression was performed for MTT assay, while two-way ANOVA followed by Tukey’s multiple comparisons test were used for migration and flow cytometer assays. Meanwhile, one-way ANOVA followed by multiple comparisons test were applied to colony survival test, oxidative biomarkers assay and the various microscopic examinations. Correlation coefficient *r* between log10 fluorescence intensity of Mitotracker Red and optical density of caspase-3 IHC protein expression was measured by Pearson’s method using RStudio (R 4.3.2).

## Results

### Preparation and characterization of optimized and stable THC-phytosomes

In the current work, three THC-phytosomes (P1-P3) were prepared at different stoichiometric ratios of THC: SPC using the thin-film hydration method (Table 1). Physicochemical characteristics, colloidal properties, and release profiles determined the selection of the optimum formula.

Based on the solubility test, pure THC showed poor aqueous solubility (3 ± 0.01 mg %). Nevertheless, all THC-phytosomes showed significant (*p* < 0.01) improvement in THC aqueous solubility compared to its free counterpart. The formulation P1 (THC: SPC 1:1) manifested the highest increase in THC aqueous solubility (8.5-fold) among all the developed THC-phytosomes. The decrease in solubility with an increase in SPC molar ratio was similar to the decrease in silybin solubility as the concentration of SPC increased [41]. This might be due to the formation of large SPC micellar aggregates enveloping THC-free drug, leading to free drug precipitation and disruption of the association-dissociation balance of the resultant complex, decreasing THC-phytosomes’ aqueous solubility.

P_o/w_ results, they well-matched solubility results, where the values for all THC-phytosomes formulae were reduced compared to pure THC, indicating an increased THC partitioning into the aqueous phase. Comparing the three THC-phytosomes formulations, P1 exhibited the lowest P_o/w_ value, while values of P2 and P3 increased with the rise in SPC amount, which may be due to the formation of large micellar aggregates, masking the polar groups and hence increasing the lipophilic nature [34, 42].

Concerning the colloidal properties, all THC-phytosomes were in the nano-size, ranging from 317.3 ± 5.4 to 442.1 ± 16.3 nm. There was a significant increase in both sizes and polydispersity index (*p* < 0.001) with the increase of SPC concentration in P2 and P3 compared to P1. This is because excess unreacted SPC molecules aggregate or self-assemble as multilamellar nanovesicles [43, 44].

Regarding zeta potential, all THC-phytosomes formulae showed stable negative potential (> -27 mV), owing to the negative phosphate group of the SPC. This negativity helped particle repulsion hence hindering their aggregation and promoting their colloidal stability [31, 45, 46].

**Fig. 1:**
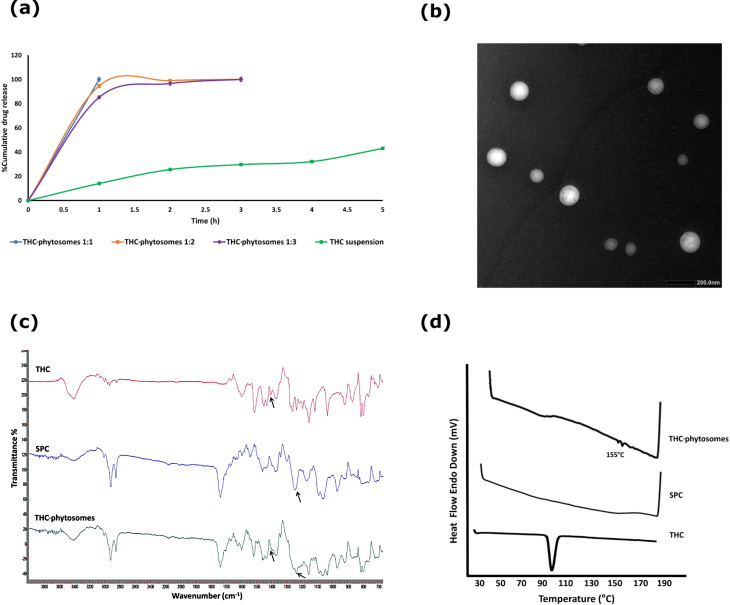
Characterization parameters of THC-phytosomes. (**a**) Release profiles of THC-phytosomes (1:1, 1:2, and 1:3) and THC suspension in 50% v/v ethanol at 37° C. (**b**) TEM of THC-phytosomes (P1). (**c**) FTIR spectrum of THC-phytosomes(P1) in comparison with THC and SPC. (**d**) DSC thermogram of THC-phytosomes (P1) in comparison with THC and SPC

As shown in Fig. 1a, cumulative drug released from THC-phytosomes was enhanced compared to THC aqueous suspension that showed only 40% drug release after five hours. On the other hand, P1 reached 100% drug release after only one hour followed by P2 and P3, which released 100% of their drug load after approximately two hours. This might be due to the amphiphilic nature of SPC in the prepared THC-phytosomes formulations that led to a higher dissolution rate because the polar head groups of the phospholipid could reduce the surface tension [45].

Based on the previous results; the P1 formulation was selected for further characterization and in vitro analyses as a phytosomal system for THC owing to its highest hydrophilicity, water solubility, and fastest drug release rate.

TEM of the selected P1 showed spherical nanosized particles with smooth surface, indicating an absence of any aggregation. Furthermore, the cores and outlines of the spherical unilamellar vesicles formed by the complex self-assembly in the aqueous solution were recognized (Fig. 1b). Per FTIR analysis in Fig. 1c, the P1 formulation spectrum revealed a broadening of the peak at 3408 cm^− 1^ corresponding to O–H group and the disappearance of a peak at 1404 cm^− 1^ corresponding to THC’s phenolic O–H associated with a shift in the PO_4_ group peak of SPC from 1242 cm^− 1^ to 1235 cm^− 1^. This might be attributed to H-bond formation between THC and SPC molecules during THC-phytosomes formation. Details in Suppl. Material.

As shown in Fig. 1d, the thermogram of THC showed a prominent endothermic peak at 100.17 °C, the melting point of THC [28]. Meanwhile, the DSC thermogram of P1 revealed the disappearance of such THC endothermic peak, with a small peak appearing at 155 °C that might be attributed to the formation of a new complex. Additionally, the difference in DSC thermogram between P1 and THC might indicate bond formation between the polar head groups of SPC and THC primarily via dipole-dipole electrostatic forces and intermolecular hydrogen bonding [47, 48]. The stability study is detailed in Suppl. Material, accompanied by Supplementary Table 1.

### Effect of THC-phytosomes on cellular metabolic activity with selectivity potential against oral cancer cells

**Fig. 2:**
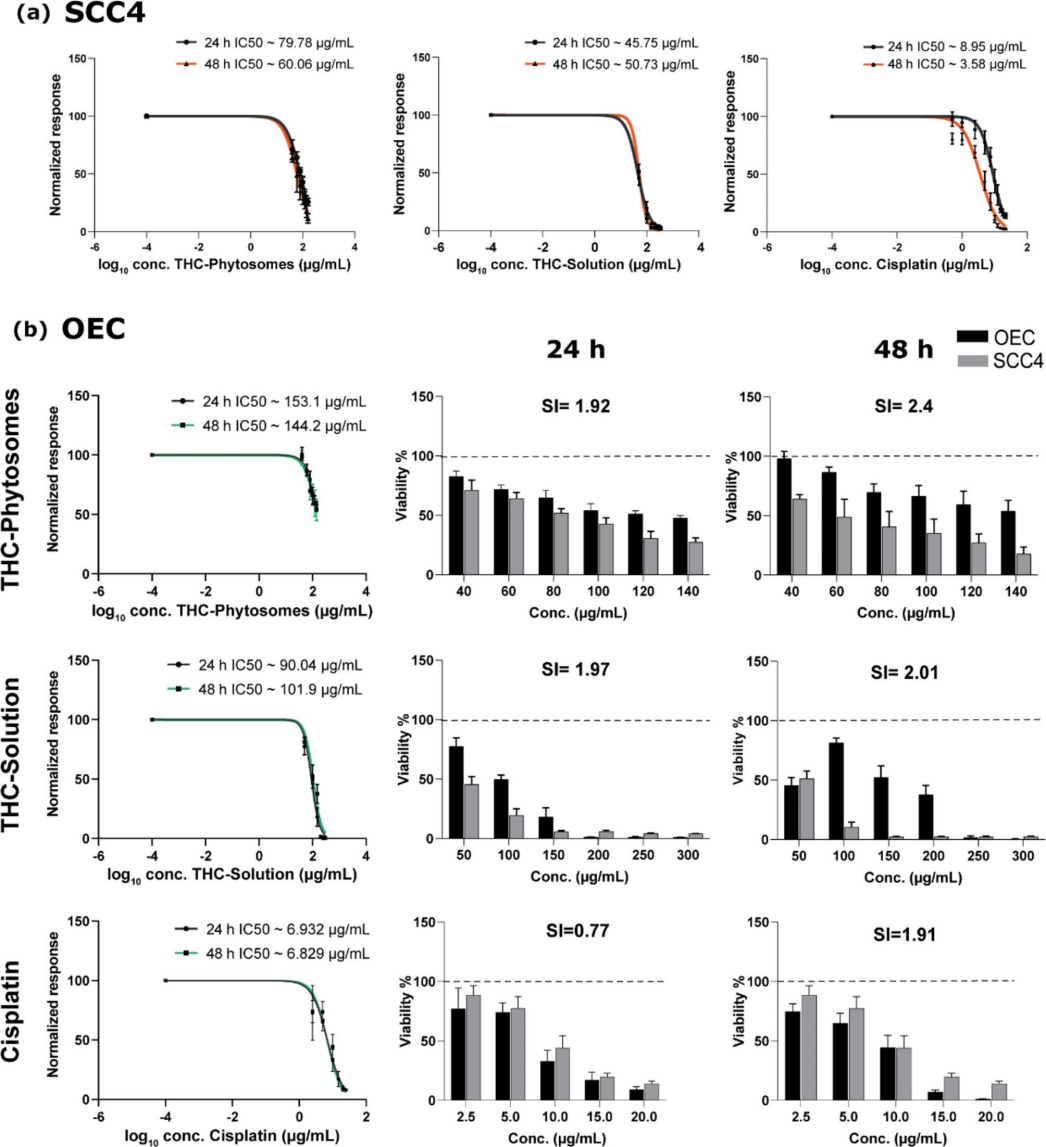
The dose-response curve of THC-phytosomes cytotoxicity on SCC4 cell line and normal oral epithelial cells. (**a**) The cytotoxic effects on cancerous SCC4 cells treated with the nanoformulation show a significant decrease in cell viability in a dose and time-dependent manner similar to the DMSO-dissolved THC-solution and the conventional chemotherapeutic agent. (**b**) The effect of the three drugs on the normal keratinocytes also shows an equivalent dose and time-dependent response, with THC-phytosomes having the highest calculated selectivity indices to oral cancer cells following 48 h of treatment. The bar charts are the mean ± SD of viability % from three independent experiments, each of triplicates in 96-well plates

As presented in Fig. 2a, the nanoformulation of the THC-phytosomes, THC-solution, and cisplatin are effectively cytotoxic, inhibiting the proliferation of SCC4 cells in a time and dose-dependent manner, thereby decreasing their viability drastically. The effective inhibitory doses of the phytosomes, solution, and cisplatin were ~ 79.78 µg/mL, ~ 45.75 µg/mL, and ~ 8.95 µg/mL after 24 h, and ~ 60.06 µg/mL, ~ 50.73 µg/mL, ~ 3.58 µg/mL after 48 h of treatment, respectively.

On isolated human cell lineages, the three medications showed a dose and time-dependent effect on normal oral keratinocytes and gingival fibroblasts. Regarding the former, the phytosomes group showed the highest selectivity index value and was the most selective to cancer cells compared with the remaining groups at 24 and 48 h intervals. It is also worth mentioning that cisplatin exhibited the least selectivity to cancer cells and caused a severe decrease in the viability of normal oral epithelial cells (Fig. 2b). Supplementary Fig. 1 in Suppl. Material shows a similar restrained toxic effect on gingival fibroblasts, tipping the selectivity towards cancer cells.

### THC-phytosomes impeded the proliferation of SCC4 cells

**Fig. 3:**
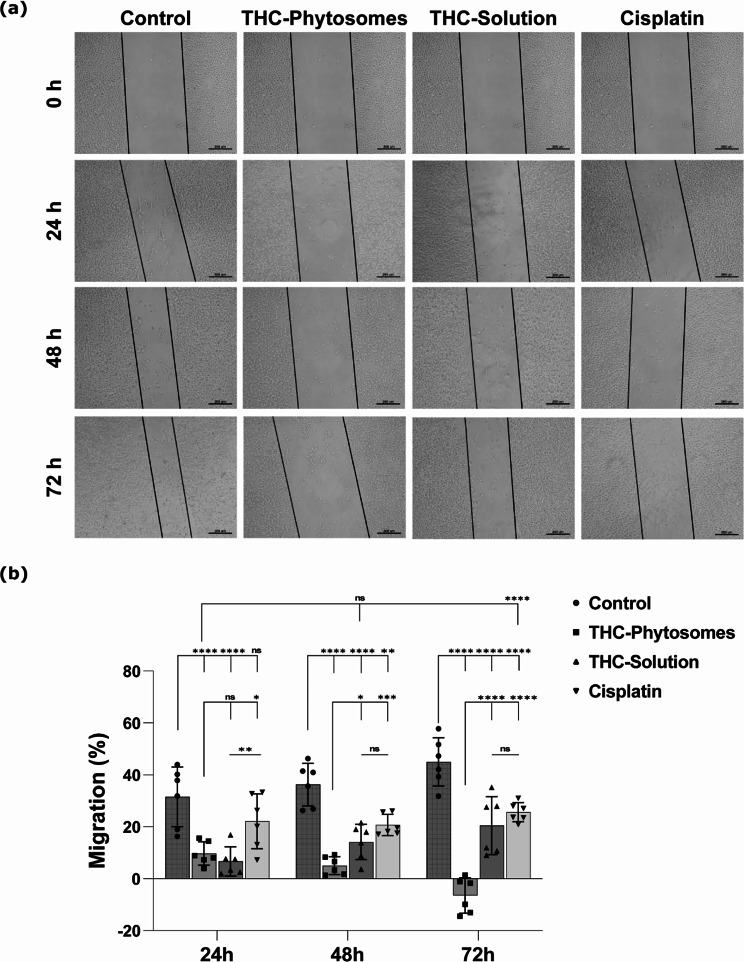
Wound closure rate evaluation of the different proposed treatments. (**a**) Photomicrographs of the scratch lines exposed to THC-phytosomes, THC-solution, and cisplatin at different timelines (24 h, 48 h, and 72 h) taking untreated oral cancerous cells as the negative control. The scratch line is the widest in the phytosomes-treated group at all time points, showing a significantly decreased migration rate percentage compared to the other proposed treatments. (**b**) A scatter plot with a bar graph representation of the migration percentage of SCC4 cells, where two-way ANOVA followed by Tukey’s multiple comparisons test reveals * of *p* < 0.05, ** = *p* < 0.01, *** = *p* < 0.001, and **** = *p* < 0.0001, while ns (non-significant) = *p* > 0.05. The data is mean ± SD of triplicates (each is a well of a 6-well plate seeded with 25 × 10^4^ SCC4 cells)

The ability of the cancerous cells to proliferate and migrate was reduced effectively in the nanoformulation of the drug compared to its original formula and the chemotherapeutic. In wound closure assay, the scratch line became progressively wider in the treated groups through 24, 48, and 72 h, recording the widest wound gap in the THC-phytosomes group and the narrowest in its solution counterpart (Fig. 3a).

The SCC4 cell migration rate exhibited a significantly progressive and sequential reduction following exposure to ~ 60.06 µg/mL of THC-phytosomes, recording 9.7 ± 4.4, 5.05 ± 3.4, and − 6.4 ± 6.8 migration % (*p* < 0.001 vs. control 31.5 ± 11.5, 36.3 ± 8.2, and 44.9 ± 9.3) at 24, 48, and 72 h, respectively. Contrarily, cancer cells treated with cisplatin showed a higher migration rate, while those treated with ~ 50.73 µg/mL of the DMSO-dissolved THC-solution showed the highest migration rate (Fig. 3b).

**Fig. 4:**
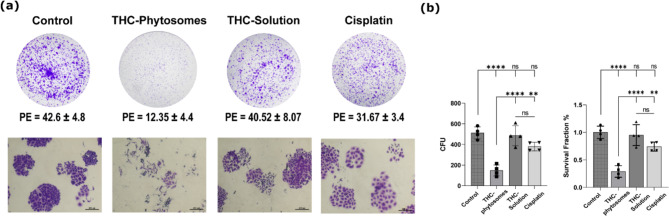
Clonogenic survival assay. (**a**) Representative photographs of cellular morphology of colonies formed after SCC4 cells treatment with phytosomes, solution, and cisplatin. (**b**) The survival fraction plot of cancer cells after administering the IC50 of the three medications, taking untreated cells as the control. A one-way ANOVA test followed by Tukey’s multiple comparisons test exhibits * = *p* < 0.05, ** = *p* < 0.01, *** = *p* < 0.001, and **** *p* < 0.0001, and ns codes for *p* > 0.05. The data is mean ± SD of triplicates (each replica is one well of a 6-well plate seeded with 25 × 10^4^ SCC4 cells)

Following treatment with the determined IC50s of the three drugs, the phytosomes group showed the highest inhibitory potential to colony formation compared with the remaining groups. The number of spheres formed was the fewest, drastically decreasing the survival fraction and plating efficiency of the nanoformulation to 0.29 ± 0.1% and 12.4 ± 4.5%, respectively compared with 42.6 ± 4.8% plating efficiency of the control (*p* < 0.0001). The THC-solution and cisplatin groups show comparable results (*p* > 0.05) that come nowhere near the phytosomes. In addition, the cellular morphology proved much worse than in the solution group or cisplatin (Fig. 4).

### THC-phytosomes promoted apoptotic DNA fragmentation, halting DNA synthesis

**Fig. 5:**
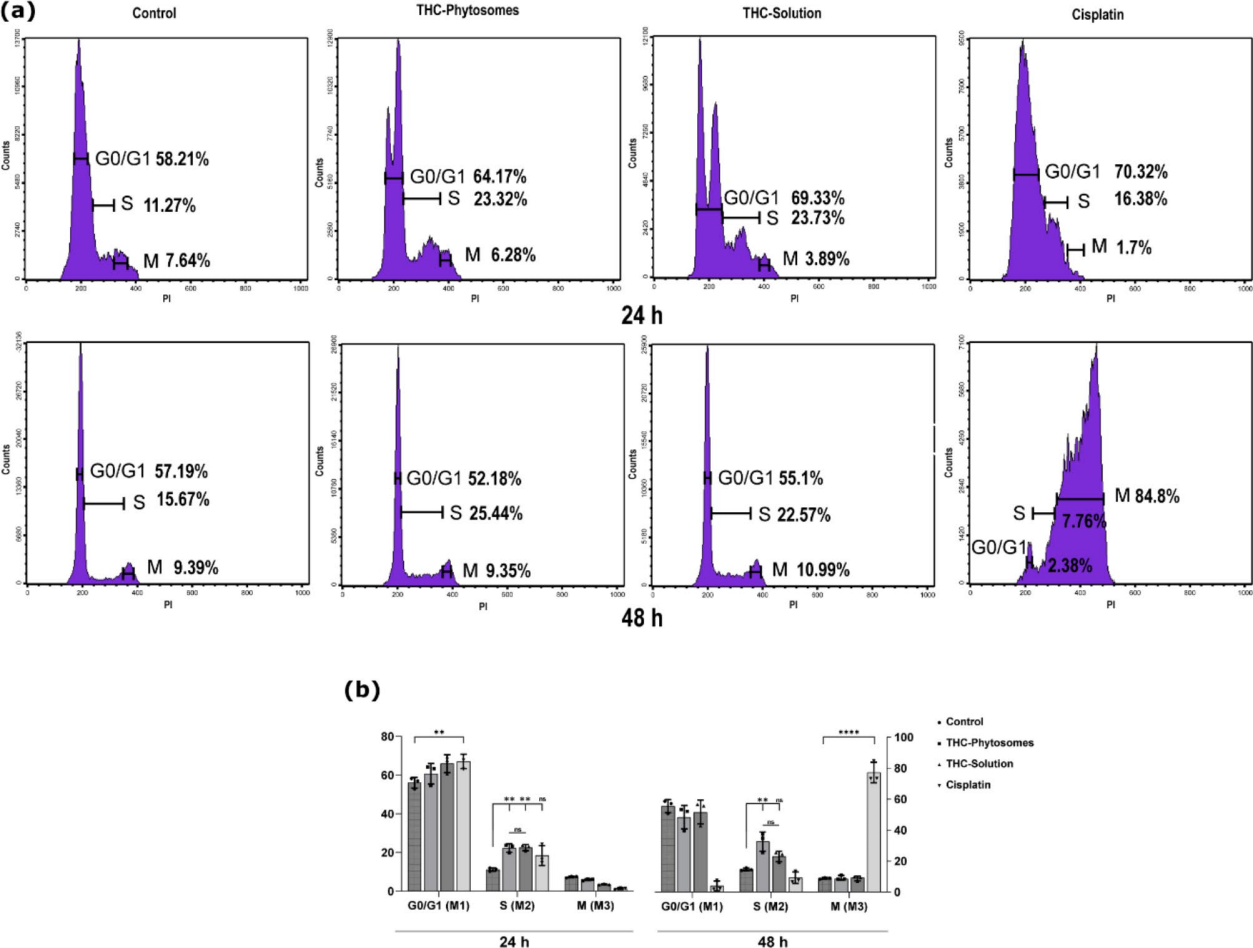
Cell cycle analysis by flow cytometry. (**a**) Representative RNase PI-marked histograms display the THC-phytosomes-induced arrest in the S-phase at 24 and 48 h. Cisplatin causes an arrest at the S-phase at 24 h, then shifts to an M-phase cell cycle arrest at 48 h. (**b**) A bar plot represents the cell population percentages in different cell cycle phases. A two-way ANOVA followed by multiple comparisons test shows * = *p* < 0.05, ** = *p* < 0.01, *** = *p* < 0.001, and **** *p* < 0.0001, while ns codes for *p* > 0.05. Data expressed as mean ± SD of triplicates (each replicate is one well of a 6-well plate seeded with 25 × 10^4^ cancer cells)

After 24 and 48 h, treated cancerous cells with the IC50 of THC-phytosomes and solution led to escalating cell percentages in the S phase with a 32.5 ± 6.22 increase compared with the control group (*p* < 0.01). The difference between the nanoformulation and the regular solution was insignificant (*p* > 0.05). However, the phytosomes showed a more time-consistent effect over the solution. Meanwhile, cisplatin halted the cell cycle in the G0/G1 phase at 24 h, then caused a shift in the cell population to the M phase at 48 h (Fig. 5).

**Fig. 6:**
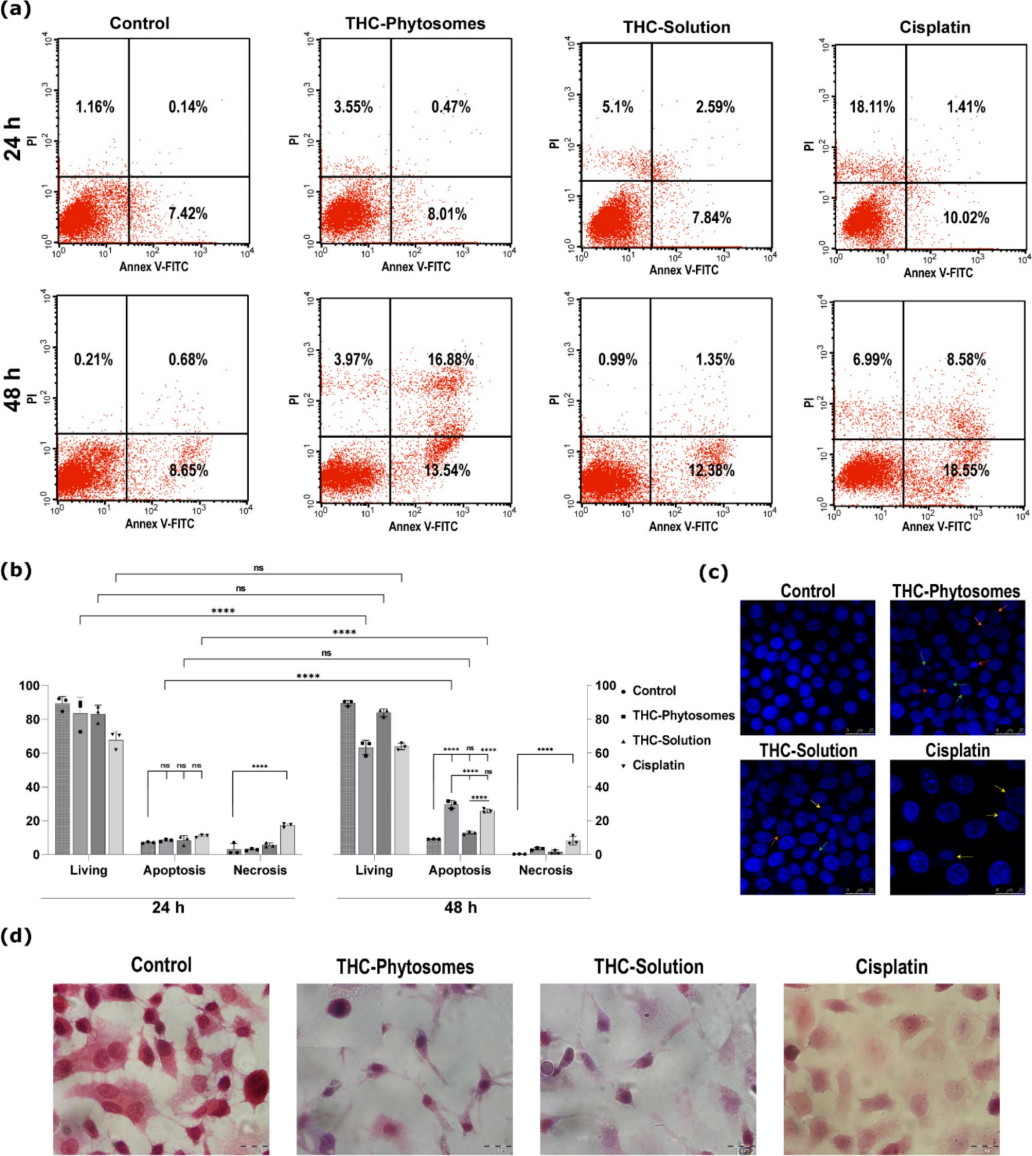
The apoptotic panel of the THC-phytosomes. (**a**) Representative annexin V/PI flow cytometer dot plots reveal that THC-phytosomes successfully enhance apoptosis in SCC4 cells at 24 and 48 h. (**b**) An interleaved scatter with bars quantifying the apoptotic cell population percentages in different proposed treatments at both time points. Data expressed as mean ± SD of triplicates (each is one well of 6-well plate seeded with 25 × 10^4^ cancerous cells), where two-way ANOVA followed by multiple comparisons test displays * = *p* < 0.05, ** = *p* < 0.01, *** = *p* < 0.001, and **** *p* < 0.0001, while ns codes for *p* > 0.05. (**c**) Hoechst nuclear-staining photomicrographs (scale bar = 25 μm) show nuclear condensation (red arrow), fragmentation (green arrow), and blebbing of the nuclear membrane (orange arrow). The apoptotic changes appear most evident in the phytosomes group. Cisplatin shows chromatin fading (yellow arrow), indicating necrosis. (**d**) H&E staining cytological images (scale bar = 20 μm) show the proliferating control cancer cells as well as multinucleated tumor giant cells, depicting the aggressiveness of the cancer behavior. Phytosomes-treated cells are undergoing apoptosis and show extreme shrinkage and apoptotic bodies (inset). With less abundance, the solution-treated group shows similar effects, while cisplatin shows sheets of ghosted cells undergoing cloudy degeneration and nuclear fading

The apoptotic flow cytometric appraisal proved the time-dependent cytotoxic profile of THC-phytosomes, which appeared to be superior to all proposed treatments in inducing SCC4 apoptotic cell death throughout 48 h of treatment with 29.69 ± 2.3% vs. 9.15 ± 0.15% of control (*p <* 0.0001), decreasing the number of living cells. Conversely, the THC-solution yielded the least effective apoptotic changes in both 24 and 48 h intervals *(p* > 0.05 compared to the control), further validating the effectiveness of the nanoformulation. On the other hand, cisplatin showed a similar apoptotic effect to the phytosomes, but had the highest count of necrotic cells compared to all groups at all time intervals (*p <* 0.0001 vs. control, Fig. 6a&b).

With Hoechst nuclear visualization, viable control cells displayed normal nuclei reflecting intense blue fluorescence. Cells treated with THC-phytosomes, and solution exhibited chromatin clumping and fragmentation, nuclear membrane blebbing, and apoptotic bodies. The apoptotic morphological changes appear most evident and intense in the phytosomes group. The cisplatin group shows evident karyolysis, where the chromatin of the nucleus is fading out, as a hallmark change of cellular necrosis (Fig. 6c).

Upon cytology, the untreated cancerous control cells appeared normal, with a polygonal shape and proper nuclear-cytoplasmic ratio. Dividing cells and multi-nucleated tumor giant cells were also observed. However, after treatment with THC-phytosomes and solution, there was a noticeable decrease in cell numbers. Phytosomes-treated cells appeared severely shrunken and had apoptotic bodies. The solution-treated cells showed shrinkage and the presence of ghost cells with chromatin clearing. In the cisplatin group, there were sheets of ghosted necrotic cells undergoing karyolysis and cloudy degeneration (Fig. 6d).

### THC-phytosomes induced mitochondria-mediated apoptosis via caspase cascade activation

**Fig. 7:**
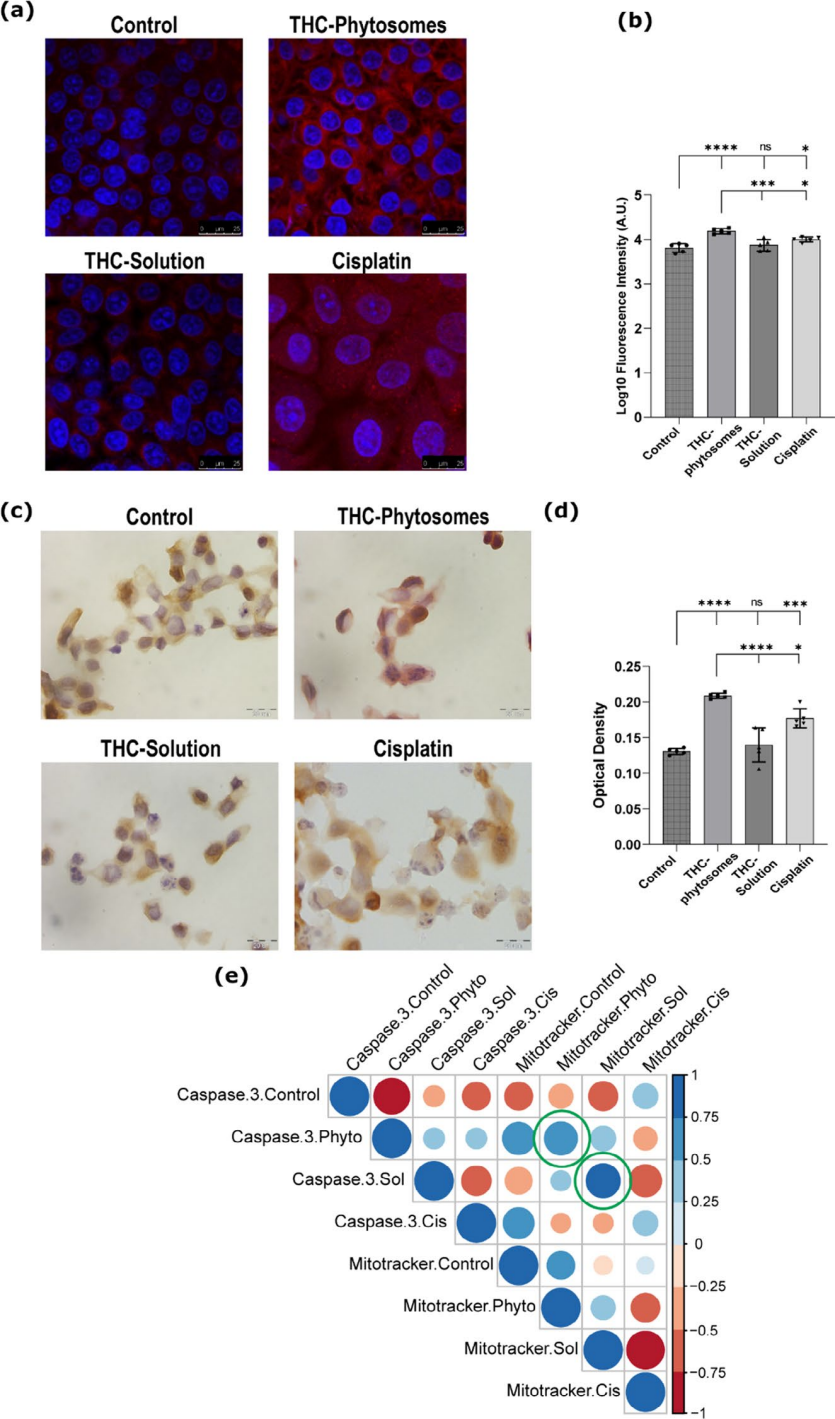
THC-phytosomes induce mitochondria-dependent apoptosis. (**a**) Mitotracker Red-staining micrographs (scale bar = 25 μm) show the most intense fluorescence in the phytosomes group, indicating mitochondrial swelling and fission, followed by the solution group. (**b**) A graphical representation of the fluorescence intensity emitted by the cells after Mitotracker Red staining. (**c**) IHC stained photomicrographs (scale bar = 20 μm) show the highest positivity of caspase-3 antibody in the phytosomes group with cytoplasmic homing of the stain and nuclei showing nuclear fragmentation, characteristic of apoptosis. (**d**) A quantitative graphical representation of the optical density displayed by the up taken stain in the different groups. (**e**) Heat map illustration of correlation matrix shows varying degrees of positive correlation between log_10_ fluorescence intensity of Mitotracker Red and the optical density of caspase-3 protein expression by IHC among all treated groups. The green circles signal out the significant positive correlation present in both THC cohorts. A one-way ANOVA test followed by Tukey’s test shows * = *p* < 0.05, ** = *p* < 0.01, *** = *p* < 0.001, and **** *p* < 0.0001, and ns codes for *p* > 0.05. The data is the mean ± SD of five random microscopical fields from triplicate wells of 6-well plate seeded with 25 × 10^4^ SCC4 cells. Correlation coefficient *r* was measured using Pearson’s method

We stained the SCC4 cells with MitoTracker Red, a fluorescent dye, to observe the mitochondrial morphology during treatment with phytosomes. The intensity and distribution of the stain allowed us to describe the morphology and integrity of the mitochondrial structure [49]. Control SCC4 cells showed unremarkable fluorescent intensity since the cells were not undergoing apoptosis. Nevertheless, cells treated with 60.06 µg/mL of THC-phytosomes displayed remarkable fluorescence, reflecting the intense mitochondrial swelling and activating the mitochondria-mediated apoptosis (Fig. 7a&b).

Caspase-3 protein showed a similar increased immune profile linked to the intensity of fluorescent-tagged mitochondria. The immunocytochemical caspase-3 showed cytoplasmic homing in all four groups. The three treated groups showed multiple cells with a morphological picture consistent with apoptosis displaying varying degrees of caspase-3 positivity (Fig. 7c). The phytosomes group showed the highest optical density and positivity to caspase-3, the effector apoptotic protein, conclusively highlighting the group’s superiority (*p* < 0.0001 vs. control). The THC-solution and cisplatin revealed comparable immuno results (*p* < 0.05, Fig. 7d).

Correlation matrix indeed showed varying degrees of positive correlation between the Mitotracker fluorescence and caspase-3 expression among all treated groups, which gives insight about the interplay between both parameters. The distinguished fluorescent Mitotracker stain was strongly correlated with the caspase-3 immunostain in the THC-phytosomes group (*r* = 0.73, *p* = 0.0063) and THC-solution group (*r* = 0.77, *p* = 0.0008), while it was of insignificant correlation in cisplatin-treated cells (*r* = 0.4, *p* = 0.1, Fig. 7e).

**Fig. 8:**
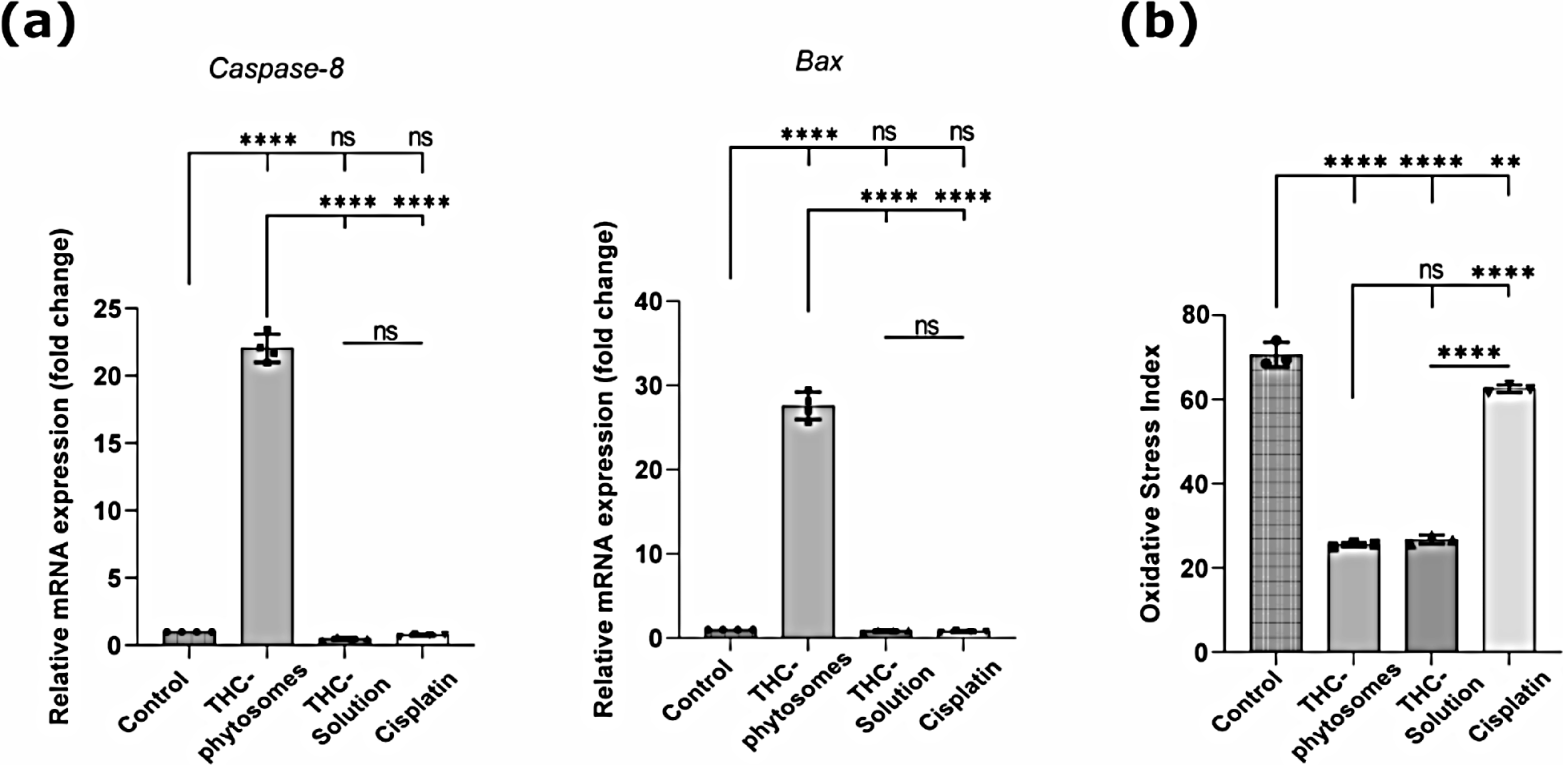
RTqPCR analysis and oxidative stress biomarkers assay. (**a**) Bar plots for RTqPCR analysis show the unparallel effect of THC-phytosomes compared with other groups in expression of *caspase-8* and *Bax*, apoptotic genes. (**b**) Another bar graph shows both THC groups culminating the lowest MDA/TAC oxidative stress index of the different test groups compared with the control, highlighting their superior radical-scavenging potential. A one-way ANOVA test followed by Tukey’s test shows * = *p* < 0.05, ** = *p* < 0.01, *** = *p* < 0.001, and **** *p* < 0.0001, and ns codes for *p* > 0.05. The data is the mean ± SD of triplicate wells of 6-well plate seeded with 25 × 10^4^ SCC4 cells

In a further elucidation of the apoptotic pathway, PCR revealed that both *caspase-8* and *Bax* expressions were singularly highest in the phytosomes group (*p* < 0.0001) and yielded a non-significant induction in the THC-solution and cisplatin groups when compared with the control (*p >* 0.05, Fig. 8a). The results prove the effectiveness of the nanopreparation versus its regular counterpart and the conventional chemotherapeutic drug in activation of *caspase-8*, the initiator protein of the apoptotic pathway, and then resuming the cascade with *Bax* expression, released later during the apoptotic event.

### THC-phytosomes effectively scavenged free radicals

After calculating the oxidative stress index, both THC groups showed an extremely potent antioxidant potential that far surpassed that of cisplatin, yielding the lowest MDA/TAC ratio (*p* < 0.0001). There was no significant difference between the phytosomes formulation and the solution (*p* > 0.05, Fig. 8b).

## Discussion

THC, an isolated major metabolite of curcumin, is a polyphenolic compound that exhibits improved bioavailability over curcumin [11]. It possesses exemplary chemo-preventive and anticancer potential [14]. Despite its advantages over curcumin in terms of chemical stability [20]. THC shows low aqueous solubility, limiting its bio-accessibility [21]. Therefore, several drug delivery systems have been created to enhance its efficiency and solubility.

Phytosomes (phyto-phospholipid complexes) are lipid-based organic delivery systems that can increase the absorption and stability of poorly soluble phytochemicals [29]. Having a high affinity for polyphenols, they can create hydrogen bonds between their polar heads and the polar part of the active ingredients they carry [50]. These bonds enhance the efficiency of drug entrapment, water solubility, and minimizes drug leakage. This, in turn, improves drug stability, increases membrane absorption, conclusively maximizing bioavailability [31]. There are several phytosomal preparations of a wide range of phytochemicals. Those with the most positive clinical evidence are curcumin and silybin phytosomes, having been the most commonly prepared [51]. Most phytosomal curcuminoids behave more superiorly than free curcuminoids, showing enhanced cytotoxicity and increased cellular uptake [52]. In another context, the nanoformulation of polyvinyl pyrrolidone-stabilized curcumin has previously shown a preferential uptake to SCC4 over normal cell lineage [9].

Hence in our present work, we aim to develop and characterize THC-phytosomes as an efficient nanoformulation. We also aim to ascertain its cytotoxic efficacy and selectivity against SCC4 cell line compared to its free solution counterpart and the conventional chemotherapeutic drug, cisplatin.

Several studies attest to the dose and time-dependent cytotoxic effect of THC on treatment-resistant cancers [17, 53]. The decrease in IC50 over time could insinuate the increased cellular uptake of the nanoparticles by endocytosis, evading phagocytosis and being more penetrative to cancer cells [54]. Meanwhile, THC-solution was inconsistent in its cytotoxic profile, depicting a difficulty in cellular uptake over time, an expected finding owing to the decreased water solubility of THC. Despite the shooting increase in the sensitivity of oral cancer cells to THC-solution compared with the phytosomes, we should not neglect the toxic effect of DMSO. Regarding the nanoformulation and the standard chemotherapeutic, as the dose increased and time elapsed, the cell viability decreased drastically. Therefore, THC-phytosomes proved to have both dose and time-dependent cytotoxic responses similar to cisplatin.

Evaluating the selectivity index of each drug, our results established the THC-phytosomes to be the most selective to cancer cells, preserving the viability of normal oral keratinocytes and gingival fibroblasts at all concentrations. However high the selectivity of the solution to cancer cells was, it showed a drastic decrease in normal cell viability when dosages increased. Of all the three medications, cisplatin showed the least selectivity to cancerous cells and was the most toxic to normal cells, highlighting this as a significant side effect of cisplatin.

Studying the ability of cells to migrate and proliferate is an integral component of cancer studies. Herein, we implored the scratch assay in cell culture after treatment with the corresponding IC50s of the three medications over 72 h. The THC-phytosomes group revealed the widest wound gap, designating that the treatment was the most effective in impeding proliferation and caused the lowest percentage of migration. As for THC-solution and cisplatin, they did not inhibit the proliferation as effectively as the nanoformula, with a faster rate of closure and a comparable wound gap. Similarly, combining THC-solution with radiation on C6 cells has revealed its potency as an anti-migratory over THC alone [37].

Clonogenic survival analysis represents a metric by which we can measure cellular proliferative loss in response to treatment or injury [55]. Asserting the anti-proliferative efficacy, the nanopreparation of THC proves to have the upper hand in inhibitory potential to colony survival, producing the lowest number of spheres and recording the lowest survival fraction among all groups. THC-solution and cisplatin again show comparable pro-proliferative clonogenic responses. These anti-proliferative findings display how effective the phytosomes strategy is for allowing a successful uptake and diffusion through the cell membrane. This is attributed to the enhanced solubility and P_o/w_ of THC-phytosomes, which could be assigned to the amphiphilic nature of the developed THC-SPC complex [32, 45], in contrast to the lipophilic nature of pure THC [31]. Further supporting these results is the drug release study, which showed obvious improvement in the hydrophilicity and aqueous solubility of THC-phytosomes formulations, enhancing both the rate and the extent of their release compared to pure THC [31].

Several reports account for the ability of numerous anti-cancerous agents to halt the cell cycle at different checkpoints, eliciting cellular apoptosis [56]. Per the considerable variation among results in the literature in the cell cycle assay, THC has resulted in the accumulation of cells in the G0/G1 phase in MCF-7 and 4T1 breast cancer cell lines [17]. In another study on glioma cells, it caused a G2/M phase arrest [57]. Our results displayed an S-phase cell population arrest by THC-solution in SCC4 cells at 24 and 48 h. This is consistent with our previous results on the hamster precancerous model, where THC decreased the immune expression of cyclin D1, a cell cycle checkpoint regulator [28]. On the other hand, cisplatin showed a G0/G1 phase arrest at 24 h, similar to that described previously on leukemic cells [58]. Then, at 48 h, the cell populations shifted substantially to cause a stark arrest at the M-phase. A previous study on gastric cancer also confirms the G2/M phase arrest after 48 h of treatment by cisplatin [59].

Our study manifested that treatment with THC-phytosomes led to a significant escalation of cell percentages in the S-phase of the cell cycle. That escalation was both increasing and consistent throughout 48 h, unlike the THC-solution, whose escalation of cell percentages in the S-phase seemed somewhat decreasing over time, and unlike cisplatin with the inconsistent responses at 24 and 48 h. Halting the cell cycle at the S-phase is a character seen in numerous researched anticancer drugs [60, 61]. This S-phase arrest would prevent DNA replication, consequently preventing cell division and growth, essentially freezing the cells. That constitutes cellular stress that might induce the cell to undergo apoptosis.

In cancer management, apoptotic pathway targeting may result in cancer death, reversing drug resistance or enhancing drug sensitivity [62]. THC-phytosomes predominated the apoptotic flow cytometric analysis over 48 h of treatment. The high count of apoptotic cells complemented by the undermined necrotic response could suggest that the nanosized preparation of THC, owing to the chemical bond between the drug and the phospholipid, allows the medication to be uptaken more readily by the cells, eliciting a more intense apoptotic response with the formation of apoptotic bodies, which can then be safely phagocytosed, Moreover, this nanosized formulation would enhance the absorption and bioactivity of the free counterpart of the drug, as it has been established with different cancer drugs when compared to their respective nanoformulations that not only did the nanosized loaded-drug provide an enhanced response but also had reduced adverse effects [63].

Backing up our Annexin V-FITC cytometric results, Hoechst nuclear and H&E cytological visualizations in the phytosomes group exhibited the hallmark features of apoptosis, namely intense chromatin fragmentation, apoptotic bodies, and nuclear membrane blebbing. For instance, in our previous oral in vivo model, the nanoformulated THC mucoadhesive composite was able to regress the evolving proliferative leukoplakic lesions that showed severe dysplasia into simple white patches with minimal atypia [28].

As for THC-solution, it showed the weakest apoptosis-inducing effect among the groups possibly because THC has a higher molecular weight in addition to a decreased aqueous solubility, which makes its passive diffusion into the cells much harder. Similarly, THC-solution has induced apoptosis in glioma cells only through combination treatment with radiation, albeit effectively enhancing their radiosensitivity [37]. Hence the microscopic features of apoptosis in the THC-solution group were less in abundance than the phytosomes group.

The favorable result of cisplatin described in the flow cytometric analysis is not entirely without consequences since cisplatin shows the highest number of necrotic cells among all groups at all time points, which seems to be a commonly described feature of the drug in literature. Evidence has shown that cisplatin-induced necroptosis (programmed cell necrosis) could be the culprit behind its routinely presented nephrotoxicity [64]. Our microscopic visualization results indeed showed that cisplatin-treated cells exhibited distinctive features of necrosis, ranging from chromatin fading (karyolysis) to cloudy degeneration and ghosted cells. Accordingly, the cost versus benefit provided by cisplatin could be arguable.

Attempting to elucidate the rationale behind the enhanced apoptotic effect of THC-phytosomes, we had to determine the pathway tackled by apoptosis. Consequently, we stained the SCC4 cells with MitoTracker Red, whose intensity and distribution have been previously used to describe the morphology and integrity of the mitochondrial structure of HeLa cells undergoing apoptosis [49]. Herein, oral cancer cells treated with THC-phytosomes displayed a remarkably intense fluorescence that marks increased mitochondrial activity and swelling [49, 65]. A previous study on aclarubicin, a fluorescent anticancer drug, showed that its fluorescence merged predominantly with the Mitotracker Deep Red staining of the mitochondria and was relevant to its increased cytotoxicity [66]. Our results from using Mitotracker Red provide insight into the mitochondria-related signaling pathway of apoptosis.

Caspase-3 is an apoptotic executioner protein, whose high levels correlate with cells displaying morphologic features of apoptosis [67]. Immunohistochemically, we found the highest detectable positivity in the phytosomes group with cytoplasmic homing similar to that previously described in paclitaxel-treated HT29, MDA-MB231, and KB cells [68]. This finding is in agreement with Liu et al. and Han et al., who concluded that THC was effective in inducing apoptosis in hepatocellular carcinoma and breast cancer cells, respectively, by increasing caspase-3 and caspase-9 protein expression [69, 70]. Since our results have found that the fluorescence of Mitotracker Red positively and significantly correlated with caspase-3 protein expression, we can hence assume that mitochondrial signaling was implicated in inducing apoptosis of SCC4 cells by THC-phytosomes. Further validating this is the increased number of apoptotic cells and the abundance of morphological apoptotic nuclear features in the THC-phytosomes group coupled with the completely low fluorescence emitted by the proliferating untreated cancer cells. This can be attributed to the fact that the water-soluble THC-phytosomes were quite easily engulfed by the cells owing to their nanoscale, causing the cells to be saturated with the drug, hence producing an elevated response.

Tracing the apoptotic pathway back to the initiator protein, *caspase-8* gene expression was extremely high in the phytosomes-treated group. Interestingly, a similar elevated expression was attained by FLLL32, a synthetic curcumin analog, previously tested on oral cancer cell lines SCC9 and HSC-3 [71]. Widely regarded as the initiator protein responsible for the extrinsic pathway of apoptosis, caspase-8 can also trigger the intrinsic mitochondria-related pathway, suggesting an interplay between both mechanisms [72].

Bax is considerably the main pro-apoptotic protein of the Bcl-2 family. In the cancer milieu, overexpression of *Bax* decelerates tumor enlargement [73]. A PCR result retrieved from the H22-induced mice model, where THC-treated tumors revealed overexpression of *Bax*, was related to reduced malignant ascites volume, indicating the possible functional role of THC in triggering the mitochondrial pathway of apoptosis [70]. Mimicking a similar *Bax* genetic profile, our results showed an enormously high expression of *Bax* in the phytosomes group compared with the other groups. This would later cause mitochondrial membrane permeabilization, releasing small pro-apoptotic molecules such as cytochrome C [74].

Tackling another arm of cellular functional phenotyping, we aimed to verify the protective antioxidant capacity of THC by measuring the released ROS, crucial players in carcinogenesis [75, 76]. Here, we assessed the oxidative stress index by calculating the ratio of MDA, a frequent byproduct of (LPO) to the total antioxidant capacity (TAC). We chose MDA as an index of LPO because of its high levels in patients with oral and oropharyngeal cancer inversely combined with the low plasma levels of antioxidants [77]. Our results mirror the evidence in the literature about the potent and superior antioxidant effect THC possesses owing to the lack of conjugated bonds in its central chain [11, 78]. Both THC formulas showed promising results and the lack of significance between both is a true affirmation of the innate antioxidant nature of the herb itself. Cisplatin shows the highest MDA/TAC ratio, indicating the highest oxidant potential of the groups, an expected finding since most chemotherapeutic agents generate ROS to induce apoptotic damage in cancer cells [79]. Precisely because of that, this strategy poses dangerously toxic side effects to normal cells, which do not escape this therapeutic approach [80]. In contrast, an antioxidant agent can promote cellular function and immunity, while reducing the side effects of ROS-producing drugs, such as cisplatin. For example, a study has prescribed antioxidant supplements to reduce cisplatin-mediated nephrotoxicity [81], which sheds light on the possible use of including THC in anti-cancer regimens.

Finally, it is important to note that verifying our results on additional cell lines and profiling cell cycle proteins such as cyclin A1 and PCNA would be beneficial. Additionally, coupling these in vitro investigations with in vivo studies would be highly recommended to help transfer our hypothesis to the translational field.

## Conclusions

In conclusion, our combined data indicate that the developed THC-phytosomes formulation is a superior and novel nanoformula that enhanced the pharmaco-therapeutic potential of the free THC solution by improving aqueous solubility and cellular uptake. Moreover, it exhibits strong toxicity against oral cancer cells while exercising a cytoprotective effect on normal cells, suggesting potential effectiveness in tissue-targeted therapy. THC-phytosomes target various molecular pathways, such as impeding cancer cell proliferation, arresting the cell cycle, promoting apoptosis, and scavenging free radicals. Our findings showed that THC-phytosomes behaved in a manner equal to or even better than one of the most commonly prescribed chemotherapeutic drugs. Therefore, they could be considered a potent anticancer agent that can be further tested on different animal carcinogenic models to transfer its efficacy potential to the translational side either on its own or alongside chemotherapeutics to help minimize their required dose and side effects.

## Electronic supplementary material

Below is the link to the electronic supplementary material.

Supplementary Material 1

## Data Availability

Data is provided within the manuscript or supplementary information files.

## Abbreviations

ATCC: American Type Culture Collection
DMEM: Dulbecco’s Modified Eagle Medium
DMSO: Dimethyl Sulfoxide
DSC: Differential Scanning Calorimetry
FTIR: Fourier Transform Infrared Spectroscopy
IHC: Immunohistochemistry
LPO: Lipid Peroxidation
MDA: Malondialdehyde
OSCC: Oral Squamous Cell Carcinoma
(Po/w): Partition Coefficient
PBS: Phosphate-buffered Saline
PE: Plating Efficiency
ROS: Reactive Oxygen Species
RT-qPCR: Reverse Transcription Quantitative Polymerase Chain Reaction
SPC: Soy Phosphatidylcholine
TAC: Total Antioxidant Capacity
TEM: Transmission Electron Microscopy
THC: Tetrahydrocurcumin
