# Supplementary material for: The anticancer potential of tetrahydrocurcumin-phytosomes against oral carcinoma progression
Source: BMC Oral Health. 2024 Sep 26;24:1126. doi: 10.1186/s12903-024-04856-9 (PMC11430579; doi:10.1186/s12903-024-04856-9)
Supplement: Supplementary file 1 — Supplementary Material 1 [file 12903_2024_4856_MOESM1_ESM.docx]

**The anticancer potential of tetrahydrocurcumin-phytosomes against oral carcinoma progression**

Nehal Raouf, Zeinab Elsayed Darwish, Omneya Ramadan, Hebatallah S. Barakat, Shimaa A. Elbanna, Marwa M. Essawy

This document contains additional information regarding the materials and methods and the results sections, in addition to an additional figure and table.

**Materials and Methods**

**Drugs and Reagents**

Soy phosphatidylcholine (SPC) (lipoid® S100) was obtained from Lipoid GmbH (Ludwigshafen, Germany). Tetrahydrocurcumin (THC) (98%) was purchased from Carbosynth Ltd. (Compton, UK). Absolute ethanol, methanol, and methylene chloride were obtained from ADWIC (Cairo, Egypt). Phospholipids were purchased from (Lipoid Co., Ludwigshafen, Germany). The following cultivating materials were obtained from Gibco, USA; Dulbecco’s modified Eagle’s medium (DMEM), Fetal bovine serum (FBS), Phosphate-buffered saline (PBS), Antibiotics (penicillin and streptomycin) and EDTA trypsin. MTT 3-(4, 5-dimethythiazol- 2-yl)-2, 5-diphenyltetrazolium bromide was purchased from SERVA-Electrophoresis GmbH, Germany. Annexin V-FITC and propidium iodide (PI) analysis kits were purchased from Sigma, USA. Dimethyl sulfoxide (DMSO) was obtained from Fisher Chemical (Fisher Scientific), UK. Caspase-3 antibody (Cell Signaling, #9662). MitoTracker Red (Molecular Probes, Invitrogen, ThermoFisher Scientific, M7512). Hoechst 33342 (Life Technologies, USA). Reactive oxygen species (ROS) kits and antioxidant assays were supplied by Biodiagnostics, Egypt. All other chemicals were of analytical grade.

**Cytotoxicity and selectivity index**

The cell viability (%) was calculated using the following formula:

$$Cell viability=\frac{Optical Density (OD) of the samples}{OD of the controls}\times100\%$$

OD of the control and OD of the sample represent the absorbance of cells cultured without or with treatment, respectively. The 50% inhibitory concentration (IC50) values of the three drugs were calculated using GraphPad Prism version 9.5.1.

The selectivity of each compound to SCC4 cells was calculated using the following formula:

$$Selectivity index (SI)=\frac{IC50 for normal cell line}{IC50 for cancer cell line}$$

**Clonogenic survival assay**

We calculated the plating efficiency (PE) and survival fraction (SF) were calculated using the following formulas:

$$PE=\frac{Number of colonies formed}{Number of seeded cells} \times100\%$$

$$SF=\frac{Number of colonies formed after treatment}{Number of seeded cells x PE of untreated cells} \times100\%$$

**Wound healing assay**

Migration rates were calculated using the following equation:

$$Migration rate\left( \% \right)=\frac{initial distance-final distance}{initial distance} \times100\%$$

The initial distance designated the width of the gap at 0 h. The final distance referred to the wound gap after 24, 48 and 72 h incubation with the proposed treatments. Analysis was done using ImageJ (1.54f, NIH, USA).

**Mitochondrial assessment during apoptosis**

Cancer cells were plated in 6‐well glass bottom plates at a concentration of 2 × 10^5^ cells/well in 2 ml growth medium and were allowed to attach overnight. After adding 100 nM MitoTracker Red, the cells were incubated for 1 h in a CO_2_ incubator at 37°C. Prior to fluorescence imaging, nuclear staining was done, where treated cells were fixed with 4% paraformaldehyde for 10 min at room temperature, washed three times with PBS, permeabilized in Triton X-100 (0.2%) for 10 min, washed three times with PBS, and incubated with 1 μg/ml Hoechst 33342 for 10 min. Visualization was done using confocal microscope Leica DMi8 (Leica, Wetzlar, Germany) equipped with a 63x oil immersion lens. The images were captured and analyzed using ImageJ (1.54f, NIH, USA).

**Reverse transcription quantitative polymerase chain reaction (RTqPCR)**

***Isolation and reverse transcription of RNA***

After forming a confluent monolayer of cells in 6-well plates, the cells were washed twice with ice cold PBS and lysed with 500 μL Qiazole. Total RNA was isolated using spin protocol (Qiagen RNeasy Mini Kit #74104). RNA concentrations and purity were detected on a Thermo Scientific ND2000 Nanodrop Spectrophotometer and stored at -80 °C. A high-capacity cDNA reverse transcription kit (Life Technologies #4374966) was used for reverse transcription of the total RNA (12.5-25 ng/μL) on a thermal cycler (Applied Biosystems SimpliAmp PCR System A24811). cDNA was stored at -20 until qPCR experiments.

***Real time PCR and data quantification***

cDNA (5-10 ng/μL) was amplified with assay primers (100 nM each; Biosearch Technologies (Novato, CA, USA) and Maxima SYBR Green/ROX kit (Thermo Scientific #K0221) on a Rotor-Gene Q, Qiagen 900163. Using the relative quantification (ΔΔCt) method, analysis of gene expression was performed with Qiagen Q-Rex Software. Results were displayed as fold change relative to HPRT (2 ^-ΔΔCt^).

**Oxidative stress biomarkers assay**

Oxidative stress was assessed in the culture media by measuring the ratio of the lipid peroxidation product malondialdehyde (MDA) to total antioxidant capacity (TAC) after administering the IC50s of the three medications. MDA was measured using the thiobarbituric acid (TBA) spectrophotometric method and the colored product was measured at 534 nm. TAC was detected by the reaction of antioxidants in the sample with a defined amount of exogenously provided H_2_O_2_. The resultant-colored product was measured at 505 nm. The ratio of MDA/TAC was calculated by dividing the MDA (nmol/mL) value by the TAC (mM/L).

**Results**

***Fourier Transform Infrared Spectroscopy (FTIR)***

By imploring FTIR analysis, the THC IR spectrum shows that the hydroxyl group (O–H) exhibited a broad absorption band at 3408 cm^-1^, C–H^sp2^ at 3064 and 3003 cm^-1^, and C–H^sp3^ at 2961, 2933, and 2844 cm^-1^. The vibrations of the carbonyl bond (C=O) were identified by a strong band at 1595 cm^-1^, and the keto-enol tautomerism of THC was identified by a tiny shoulder at 1664 cm^-1^. Medium absorption bands at 1449 cm^-1^ for C–H bending of the methyl groups, 1404 cm^-1^ for O–H bending, and 1113 cm^-1^ for C–OH were present. A strong, sharp vibrational band at 1509 cm^-1^ for aromatic ring C=C stretching, 1262 cm^-1^ for enol C–O stretching, and 1031 cm^-1^ for –C–O–CH_3_ stretching. SPC spectrum exhibited characteristic peaks at 1733 cm^−1^ and 1242 cm^−1^, which were attributed to C=O stretching and P–O asymmetric stretching, respectively. The strong peaks at 2922 cm^−1^ and 2852 cm^−1^ and the weak peak at 1374 cm^−1^ in SPC could be due to stretching and deformation of methyl groups. The peak at 1457 cm^−1^ observed in SPC could be due to bending vibration of CH_2_. While the peak at 1089 cm^−1^ referred to C–O–P symmetric stretching. Additionally, the band at 967.4 cm^−1^ might be attributed to N^+^(CH_3_)_3_ stretching (1).

***Stability study****:* The colloidal properties stability of P1 was evaluated at one-month intervals for 4 months at 4°C after hydration. As shown in Supplementary Table 1, there was non-significant change (*p* > 0.05) in PS and ZP. However, there was a significant increase in PDI values but still less than 0.5. These findings suggest a homogeneous size distribution, along with an absence of aggregation during the storage period at 4°C indicating good physical stability of P (2). This could be explained by the fact that nanosystems should have a surface charge of about ± 25 mV to keep their colloidal stability. The selected formulation P1 remained stable for 4 months because it maintained its zeta potential value within the acceptable negative range. This resulted in a strong repulsion force between the vesicles preventing them from aggregating and, as a result, giving P1 a high stability (3, 4).

**Supplementary file references**

1. Zhang J, Tang Q, Xu X, Li N. Development and evaluation of a novel phytosome-loaded chitosan microsphere system for curcumin delivery. International Journal of Pharmaceutics. 2013;448(1):168-74.

2. Tafish AM, El-Sherbiny M, Al‐Karmalawy AA, Soliman OAE-A, Saleh NM. Carvacrol-Loaded Phytosomes for Enhanced Wound Healing: Molecular Docking, Formulation, DoE-Aided Optimization, and in vitro/in vivo Evaluation. International Journal of Nanomedicine. 2023;Volume 18:5749-80.

3. El-Menshawe S, Ali A, Rabeh M, Khalil N. Nanosized soy phytosome-based thermogel as topical anti-obesity formulation: an approach for acceptable level of evidence of an effective novel herbal weight loss product. International Journal of Nanomedicine. 2018;Volume 13:307-18.

4. Peanparkdee M, Yooying R. Enhancement of solubility, thermal stability and bioaccessibility of vitexin using phosphatidylcholine-based phytosome. NFS Journal. 2023;31:28-38.

**Figure, figure legend, and table**

**
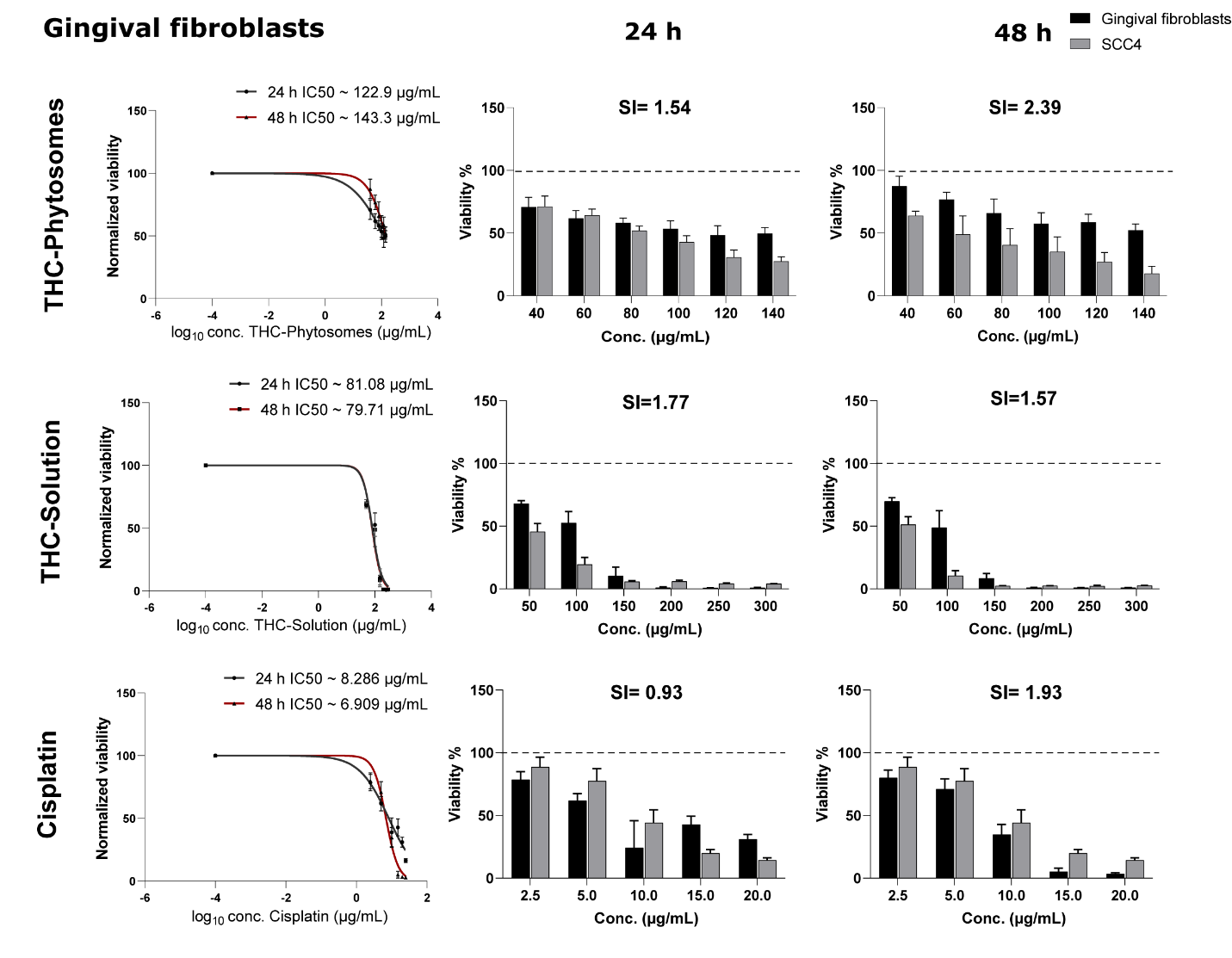
**

**Supplementary Figure 1.** **The dose-response curve of THC-phytosomes cytotoxicity on gingival fibroblasts.** The effect of the three drugs on gingival fibroblasts shows an equivalent dose and time-dependent response, with THC-phytosomes having the highest calculated selectivity indices to oral cancer cells after 48 h of treatment. The bar charts are the mean ± SD of viability % from three independent experiments, each of triplicates in 96-well plates.

**Table**

| Time (months) | Particle size (nm) | Polydispersity index | Zeta potential (mV) |
| --- | --- | --- | --- |
| Zero | 317.38 ± 5.48 | 0.285 ± 0.03 | -29.88 ± 0.351 |
| 1 | 320.38 ± 7.87 | 0.425 ± 0.07 | -28.35± 0.245 |
| 2 | 316.35 ± 6.19 | 0.376 ± 0.03 | -30.24 ± 0.337 |
| 3 | 325.87 ± 4.53 | 0.432 ± 0.04 | -29.57 ± 0.532 |
| 4 | 323.05 ± 9.87 | 0.443 ± 0.03 | -28.48 ± 0.442 |

**Supplementary Table 1. Stability data of THC-phytosomes formulation (P1) during storage for 4 months** **at 4°C**.
